# Association between a 50bp Ins/Del polymorphism at the promoter region of the superoxide dismutase-1 and age of onset of schizophrenia

**DOI:** 10.17179/excli2019-1030

**Published:** 2019-04-01

**Authors:** Niloufar Mirsadraee, Mostafa Saadat

**Affiliations:** 1 Department of Biology, College of Sciences, Shiraz University, Shiraz 71467-13565, Iran

## ⁯⁯

***Dear Editor,***

Numerous studies suggested that oxidative stress is implicated in the development of schizophrenia. Superoxide dismutases (SODs; EC 1.15.1.1) defend against cellular toxicity by metabolizing highly reactive superoxide radicals into O_2_ and H_2_O_2_ which are less reactive molecules. Superoxide dismutase-1 (SOD1, OMIM: 147450) is a cytosolic enzyme that contains copper and zinc in its active site (Zelko et al., 2002[10]).

Numerous genetic variations have been identified in the *SOD1*, including a 50bp Insertion/Deletion (Ins/Del) polymorphism in the promoter region of the gene (1684 bp upstream of the ATG start codon). Studies have been reported that the Del allele reduces promoter activity of the gene (Broom et al., 2008[1]; Saify and Saadat, 2017[8]). It has been reported that although this polymorphism is not associated with the risk of bipolar disorder type 1, it is associated with age of onset of the disorder (Kordestanian and Saadat, 2017[2]). The *SOD1 *gene is located to band q22 of human chromosome 21 (Sherman et al., 1983[9]), a region which is associated with the schizophrenia risk (Maziade et al., 2001[4]). Taken together, we hypothesized that the *SOD1* Ins/Del genetic variation might be associated with the schizophrenia risk. Therefore the present case-control study was carried out in Shiraz (Iran).

Using the Quanto software, to identify a significant difference in genotypic frequency between the cases and controls with a power of 0.80, α=0.05 (two sided), minor allele frequency=0.15, R_G_=1.50, and additive mode for inheritance; a minimum of 329 subjects would be necessary in each group. This study included 363 (268 males, 95 females) schizophrenia patients and 361 (266 males, 95 females) healthy blood donors as controls. The schizophrenia patients and healthy controls were recruited for the study as described previously (Mazaheri and Saadat 2015[3]). We lost DNA samples of 2 control subjects. The two groups were frequency matched based on age and gender. Because of heterogeneity in Iranian population (Rafiee et al., 2010[6]; Nasseri et al., 2015[5]), we selected both groups from Persian (Caucasians) Muslims living in Fars province (Iran). Written informed consents were obtained from all participants. The study was approved by the Ethics Committee of Shiraz University. Genotyping was carried out using specific primers as described previously (Saify and Saadat, 2017[8]). 

The Ins/Ins, Ins/Del and Del/Del genotypes were observed in 268, 86, and 7 of healthy controls and 275, 83, and 5 among schizophrenia cases, respectively. The genotypic frequencies in controls were consistent with the Hardy-Weinberg equilibrium (χ^2^=0.01, df=1, P=0.975). Odds ratios (ORs) and 95 % confidence intervals (CIs) were calculated to determine the association between the *SOD1* genotypes and the risk of schizophrenia. The Ins/Ins genotype was assumed as reference group (OR=1). No association was observed between the Ins/Del genotype (OR=0.94, 95 % CI=0.66-1.32, P=0.728) and Del/Del genotypes (OR=0.69, 95 % CI=0.21-2.22, P=0.540) with the risk of schizophrenia.

**Figure 1:**
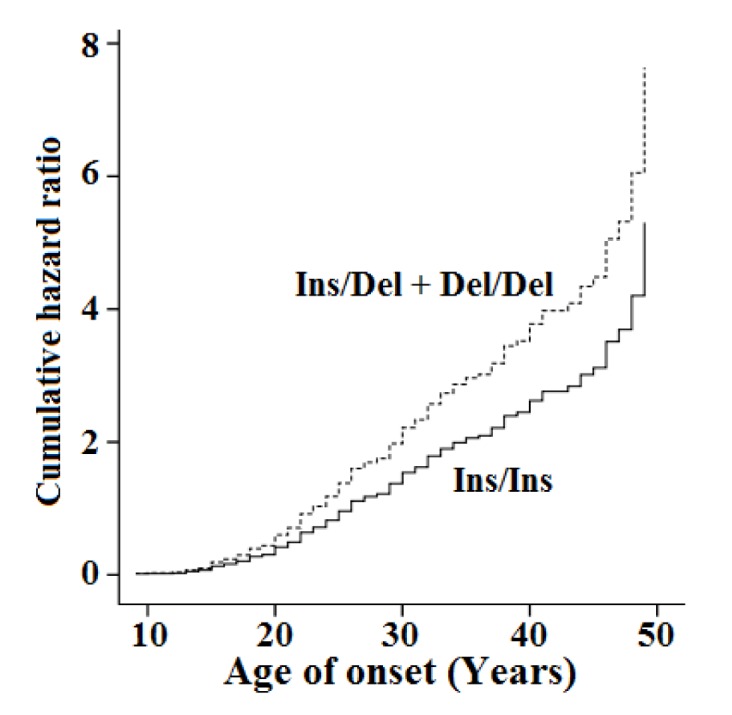
Association between the *SOD1* Ins/Del polymorphism and age of onset of schizophrenia

To investigate the relationship between the study polymorphism and age of onset of schizophrenia, the Cox proportional model was used. Age of onset of symptoms was collected from the medical records of the patients. In Cox proportional hazards regression model, schizophrenia was defined as event and age of onset was included in the analysis as time period to event. The median (mean ± SE) age of onset of the Ins/Ins and carriers of the Del allele was 23.0 (25.0 ± 0.62) and 21.0 (21.7 ± 1.09) years, respectively. Statistical analysis revealed significant association between age of onset and the genotypes of the *SOD1* Ins/Del polymorphism (Figure 1(Fig. 1)). The age of onset of schizophrenia was significantly lower in the carriers of the Del allele than the Ins/Ins genotype (Hazard ratio=1.44, 95 % CI: 1.06-1.95, P=0.019).

Previously it has been reported that there was no association between an Ins/Del polymorphism in 3^rd^ intron of the *XRCC4* and the risk of breast cancer, whereas this polymorphism showed significant association with the age of onset of breast cancer (Saadat and Saadat, 2015[7]). Analogous findings were reported on relationship between the *SOD1* Ins/Del genetic polymorphism and the risk of bipolar disorder type 1 and age of onset of the disease (Kordestanian and Saadat, 2017[2]). The present study reveals similar findings. Taken together, it seems that susceptibility to schizophrenia and the age of onset of schizophrenia are different traits.

Considering that the magnitude of the alteration of *SOD1* enzyme activity and its mRNA levels may depend on several factors, and the fact that ethnicity may influence the associations in multifactorial complex disease, replication of this study (with larger sample size) in other populations is recommended. 

## Acknowledgements

Authors are indebted to the participants for their close cooperation. The study was supported by Shiraz University, Iran (93GRD1M1741).

## Conflict of interest

None.
